# Salinity hazard drives the alteration of occupation, land use and ecosystem service in the coastal areas: Evidence from the south-western coastal region of Bangladesh

**DOI:** 10.1016/j.heliyon.2023.e18512

**Published:** 2023-07-21

**Authors:** Rofiqul Islam, Romel Ahmed, Biplob Dey, Md. Saiful Haque, Sokina Aktar, Md Saifuzzaman Bhuiyan, Mohammad Saidul Arif, Md. Ahosan Habib Ador, Mohammed Masum Ul Haque, Narayan Saha

**Affiliations:** a Ministry of Public Administration, Dhaka, Bangladesh; b Department of Forestry and Environmental Science, Shahjalal University of Science and Technology, Sylhet, 3114, Bangladesh

**Keywords:** Salinity, LULC, Occupation, Livelihood, Ecosystem services, Mangrove

## Abstract

Understanding the salinity effects on the rural livelihood and ecosystems services are essential for policy implications and mitigations. Salinity-driven modulation in land use and land cover, community traditional occupations, and ecosystem service have been elucidated in the present investigation. The study was carried out in the south-western region of Bangladesh as a representative case using focus group discussions, questionnaire survey, and remote sensing techniques. The findings showed that salinity-induced land use changes seriously threatened ecosystem services, employment and livelihoods. Shrimp farming was found to have replaced the majority of agricultural and bare lands, which led to the poor locals losing their land. The increasing land transformation to shrimp ponds as a coping strategy with salinity was not reported to be a viable option as maximum marginal poor people were unable to run the capital-intensive shrimp aquaculture. Eventually, many rich people occupied the cropland for shrimp farming which forced the traditional farmers and fishermen to leave their job and sell their labor. Many of the traditional services derived from the ecosystems were drastically reduced or got lost. The ultimate effect on the traditional livelihoods of the communities increased vulnerability and reduced resilience. The findings could aid in formulating realistic policies and action for ensuring the future resilience of the community through an appropriate adaptation strategy, such as introducing salinity-tolerant crops and integrated farming to safeguard the interest of the poor farmers. Despite the geographical locality of the study, its implications are global given the identical salinity concerns in other emerging nations' coastal regions.

## Highlights

• Salinity-driven effects on LULC, livelihoods and ecosystem services were explored.
• Household surveys, focus group discussions and remote sensing techniques were used.
• The study revealed a drastic transformation in land use systems from 1990 to 2021.
• The changes in LULC exacerbated the occupations and livelihoods of the community.
• The findings could aid in formulating policy to increase the community resilience.

## Graphical abstract

**Figure:**
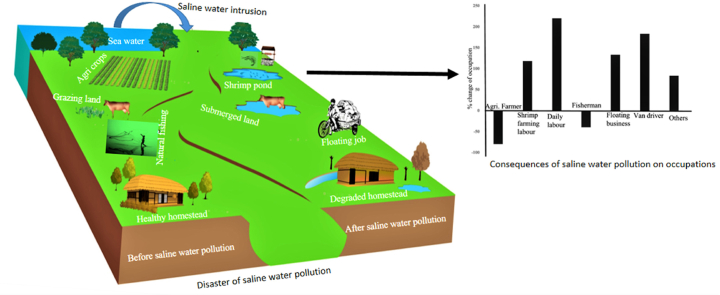

## 1 Introduction

Salinity is a major issue in Bangladesh's coastal region, which makes up 32% of the country's total land area. It is estimated that roughly 53% of the country's coastal areas are afflicted by various degrees of salinity brought on by anthropogenic and climate change [1]. A study [2] depicted a rise of soil salinity from 0.833 million ha to 10.56 million ha between the year 1973–2009 amounting to about a 0.74% increase per year affecting 3.5% of the coastal land of the country. By 2050, it is anticipated that the salinity situation in the coastal region will have gotten worse [3], leading to a loss of approximately 10% of food production [4]. The underlying reasons for increasing salinity are due to the geographic location of the country, coastal morphology, global climate change, geo-environmental politics, cross-boundary river policy and national governance. Two recent cyclones, Sidr and Aila, struck the nation in 2007 and 2009, respectively, and quickly caused a broad area to become extremely salinized [5]. The influx of salt water into coastal inland areas primarily depends on fresh watercourses that come from rivers upstream and seawater increase. Water diversions in the dry seasons by India from 25 rivers out of 54 sharply dropped the normal course of freshwater flow that allowed to intrude the seawater, the consequence is the increased salinization in the soil [5,6]. A growing body of research on the salt effect in coastal Bangladesh explores various elements of the implications on society, the environment, and the economy. For instances, salinity impact on livelihood and adaptation approaches were discussed in Refs. [1,7,8], a considerable decline in rice's yield, growth, and functional loss of physiological and biochemical properties under salt stress were reported in Refs. [[9], [10], [11], [12], [13], [14]], impact on crop diversity and food security were discussed in Refs. [15,16]. In contrast to the numerous adverse effect, salinity brings a good fortune for the people having substantial financial resources and considerable lands, they opt for shrimp farming instead of rice cultivation as the monetary return in shrimp farming is many folds higher than rice cultivation [17]. Land transformation to shrimp farms started in 1970s [15,18], has been increased to many folds, an estimate of 400% shrimp farming expansion was reported between 1984 and 2015 b y Ref. [19]. The contribution of shrimp farming to GDP, employment and income generation of the shrimp farmers are well recounted in a number of studies [[20], [21], [22]]. The profit of shrimp as alternate adaptive farming under salinity stress condition were discussed in other studies [[20], [21], [22]]. The rich landholders and outsider businessmen grabbed and occupied hundreds of acres of land for shrimp farming by converting agricultural land into shallow earthen ponds [23,24]. Natural saline water intrusion is further exacerbated by building canals for transferring saline water from the seas [23], such interventions of the shrimp farmers intensified the salinity pollution in the crop lands. The ultimate impact of salt stress is immense on a range of sectors including social, economic, and ecological contexts.

While there is an expanding body of literature on the salinity effect in the nation's coastal regions [1,7,8,16], there is still a need to update the existing literature on the impact of land alteration on livelihoods and ecosystem services. Specifically, there is a lack of discussion on community perceptions regarding the root causes of changes in their livelihoods and the link between changing occupations and the conversion of land to commercial farming. Therefore, it is necessary to explore and update the literature on the impact of land alteration on livelihoods and ecosystem services, with a focus on understanding community perceptions and the linkages between changing occupations and land use. Additionally, the big concern is the consequence of shrimp aquaculture on social and environmental hazards particularly the creation of socio-ecological challenges [25]. In spite of the suggestions of shrimp farming as an adaptive strategy under the progressively salinizing condition of the coastal areas due to lucrative profit [26], how the adoption of this coping strategy augments or aggravates the well-being of the rural communities has received less research attention in Bangladesh and elsewhere in the world. How it has marginalized the poor local inhabitants in terms of livelihoods is a focal question to be answered before advocating the shrimp farming as a viable option.

In the coastal regions of the nation, there are about 35 million residents who predominantly depend on paddy-based agriculture on 1.056 million hectares of farmed land that are subject to various levels of saline pollution [25,27]. A decrease in production due to increased salinity in the rice field [27] served as a catalyst for the conversion of the area to shrimp cultivation. Their way of life may be under danger due to the land's inability to supply essential ecosystem services and the widespread conversion of traditional subsistence agricultural fields to commercial aquaculture based on shrimp farming. A comprehensive study presenting land use changes and consequently livelihood changes are of paramount importance for the policy formulation and future adaptation. Thus, the present study was conducted to find out the key drivers of livelihood changes in the coastal districts of Bangladesh to scale up the impact of salinity among the different influential factors, to assess the land use and land-cover changes over the last 30 years, and how livelihood outcomes are changing with the changes of LULC (land use and land cover) and whether such changes consequently have affected the ecosystem services of the community living in the coastal belts.

## 2 Materials and methods

### 2.1 Study area

**Fig. 1:**
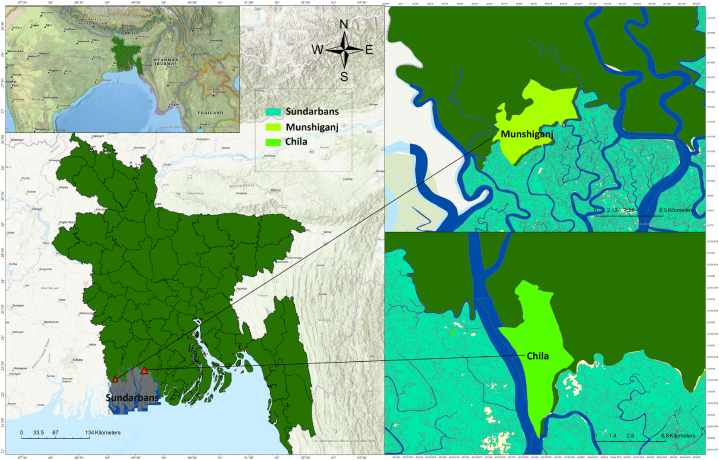
Map of the study area showing Chila union and Munshigonj union located in Bagerhat and Satkhira districts respectively of Bangladesh.

The study was conducted in the south-western region of Bangladesh which is prone to climatic disasters as well as soil and water salinity hazards (Fig. 1). Bagerhat and Shatkhira districts are recognized as moderate saline zone and high saline zone respectively [2,28]. These areas are intersected by a vast network of waterways that are influenced by tides, and very close to the Sundarban mangrove reserve forests [29]. Considering the differences of degree in salinity (Moderate and high), Bagerhat and Satkhira districts were selected purposively for the study. Following a stratified random sampling strategy, two unions (smallest administrative unit) namely Chila (located in Mongla subdistrict of Bagerhat district) and Munshigonj (located in Shyamnagar subdistrict of Satkhira district) were selected (Fig. 1). Both of the study units-Chila and Munshigonj are densely populated, density of which were recorded as close to 1100 per square kilometer [30]. The majority population of the area are Muslim (72%) followed by Hindus (27%) and small fractions are Christians other religion followers.

### 2.2 Sampling and data collection procedure

The study was conducted utilizing participatory rural appraisal (PRA), which included key informant interviews, focus group discussions (FGDs), transect walks, and one-on-one interviews with farmers. To determine the sample sizes ($S_{size}$) for the study, the number of agricultural households in the selected villages were taken into consideration for calculation. We used 95% confidence level with a 10% marginal error using Eq. (1) and also the finite correction factor employed for correcting the determined $S_{size}$ Eq. (2) based on Berenson and Levine [31]. $S_{0}$ represents the sample size before accounting for the finite household factor. Z indicates the confidence level, $h_{p}$ denotes the proportion of households, $S_{e}$ represents the sampling error, and $S_{size}$ represents the sample size after accounting for the finite household correction factor. Lastly, $N_{t}$ represents the total number of households in the study area.

(1) $$S_{0}=\frac{Z^{2}\;h_{p}\;\left(1-h_{p}\right)}{S_{e}^{2}}$$

(2) $$S_{size}=\frac{S_{0}\;N_{t}}{S_{0}+\left(N_{t}-1\right)}$$

Based on this approach, 240 respondents were selected for the interview of which 40 respondents were chosen from each of the 6 study villages in both study unions using stratified random sampling technique (Table 1). It is noted that the social and economic characteristics of the selected villages were not identical. While the villages may differ in their economic conditions, they were found to be relatively similar in terms of population size and geographical area. This similarity allows for a comparable distribution of the sample across the villages. The stratified random sampling technique was employed to create strata based on different criteria. In this case, the stratification was based on two types of households within the villages: those closely related to the local economy and holding their own land up to one acre, and those who are landless and depend on daily wage work. By including both types of households, we aimed to capture a comprehensive understanding of the socio-economic dynamics in the study area. As for the choice of uniformly selecting 40 respondents from each village, this decision was made to avoid potential bias that could arise from an imbalanced distribution of samples across villages. To make the sampling households representative with respect to their homogeneity, the choices were narrowed down to the agricultural households only for the respondent survey. To cross-check the information provided by the respondents, 4 FGDs and 6 key informants’ interviews were conducted (Table 1).

**Table 1:** Two stage sampling strategies that were adopted for conducting this study, where MSZ and HSZ refers to moderate and high saline zone respectively.

Stratified sampling					Random sampling			
Coastal zone of Bangladesh	Khulna Division	Bagerhat	Mongla subdistrict (out of 9 subdistricts) as MSZ	Chila Union (out of 6 unions) as MSZ	3 villages (out of 14)			
					Keyabunia	40	Total Respondents 120	2 FGDs and 6 Key informants
					South chila	40		
					Holdibunia	40		
		Satkhira	Shyamnagar subdistrict (out of 7 subdistricts) as HSZ	Munsigonj Union (out of 12 unions) as HSZ	3 villages (out of 20)			
					Munsigonj Bazar	40	Total Respondents 120	2 FGDs and 6 Key informants
					Dhankhali	40		
					Pankhali	40		

Before conducting the final survey, a pilot reconnaissance survey was accomplished paying a visit to the salinity-affected areas and preliminary information on the extent of salinity, livelihoods and ecosystem services were collected from the school teachers, religious leaders, elderly persons, government and non-government local officials. Based on the reconnaissance survey, a semi-structured questionnaire was prepared and all the settlers in the study areas were excluded from the interview. To obtain the historical information, only age-old persons were included, and women were also excluded from the questionnaire survey except their inclusion along with males in the focus group discussions (FGD) considering that many women are from the outside of the study area and came to the family after marriage. In each respondent survey, the objectives of the study were informed to the respondent and sufficient time was allowed to listen and note down the data.

The study areas are in proximity to the Sundarban mangrove forest (Fig. 1), has a high demand for shrimp culture instead of age-old traditional farming or fishing due to increasing progress of salinization. In context of changing the utilization pattern of the lands, these areas are ideal for assessing the ecosystem services (ES) through participatory approach. Naturally inundation and intrusion of saline water by human intervention for shrimp farming making the study area a typical characteristic of wetland with high socio-ecological complexity. Thus, a Rapid Appraisal of Wetland Ecosystem service (RAWES) approach as suggested by Ref. [32] has been used. Through local workshop and group meetings, the participants were given a clear picture of the meaning of ecosystem assets and the potential services derived from the ecosystem assets. The millennium ecosystem assessment categories were explained to the participants to make easy for the final selection of services. In the workshops and group meetings, a total of 12 services under four broad categories were selected for evaluation. The participants were from a wide range of professions, 20 were the real user of the resources derived from the land of which 10 was male and the remaining were female, 3 was the owner of the shrimp farm, 2 school teachers, 2 local forest officials, two were the non-government officials, 1 environmental activist and remaining 5 was the daily laborer of the agricultural farm or shrimp farm. All the evaluators of the ES were adults, most of them were above 45 years old. Participants were asked to put a score between 0 and 4 and ranked the values as; 4 = very high capacity, 3 = high capacity, 2 = moderate capacity, 1 = low capacity and 0 = no capacity to provide a particular services currently they derive from the ecosystem. A matrix modelling of ES was done to assess the present land use capacity to provide ES.

### 2.3 Detection of land use change

Two different types of Landsat satellites, namely Landsat 5 and Landsat 8, were used to capture images using Landsat Thematic Mapper (LTM) and Operational Land Imager (OLI) technologies. These images had a resolution of 30 m and were used to assess the changes in the land use system over a span of 30 years (1990–2021). The United States Geological Survey (USGS) provided the satellite images for four specific time periods (Table 2). 10-year intervals were chosen in our study for image analysis, because it provides a sufficient time period to capture changes in the environment while minimizing the impact of short-term fluctuations and seasonal variations. Additionally, some historical analysis has shown significant changes at similar time steps. Some other studies [33,34] also used 10 year interval for analyzing the satellite imagery. Moreover, we used satellite images in January, because it can be a good time to acquire satellite imagery due to less cloud cover and haze for fewer atmospheric distortions as well as appearance of less natural water bodies in this dry period of January. Additionally, in this season, vegetation is at its lowest point resulting in the bare ground is more visible, that is useful for land cover analyses. Moreover, during this time farmers prepare their fields for the upcoming planting season, which can be useful for agricultural analyses.

**Table 2:** List of acquired satellite images from the USGS (United States Geological Survey).

Satellite	Row/Path	Sensor	Acquisition date	Resolution (m)
Landsat 5	137/45	TM	January 21, 1990	30
Landsat 5	137/45	TM	January 19, 2000	30
Landsat 5	137/45	TM	January 30, 2010	30
Landsat 8	137/45	OLI-TIRS	January 23, 2021	30

We reprojected the satellite images to the Universal Transverse Mercator (UTM) coordinate system to make them compatible with the shapefile used for the area of interest extraction. Using a geo-referenced shape file for Chila and Munshiganj, a portion of the study site was extracted as an area of interest from each image. In diverse environmental conditions, sun azimuth and sensor reaction show the difference in the spectral response of land surface reflectance. Thus, radiometric and atmospheric correction of the images are done to normalize the image and make them comparable. As time arrangement information was used for the study, the image information was changed over into ‘at sensor reflectance’ or ‘top of atmosphere reflectance (TOA)’ information to make them operational for comparison.

The maximum likelihood classification method was used to detect the LULC classes. Training samples were prepared beforehand, and the sites were visited to get clear information on the land use at a certain location on the map. We chose five land use classes because they are the most common land use types in our study area and are relevant to our research objectives. Thus, five land use classes were classified which were settlements, agricultural lands, bare lands, shrimp farms and vegetation. Here, settlements covered all the infrastructures in the study area which included residential (homestead), commercial, agricultural, roads, culverts, and bridges. Besides, farmlands and grazing lands were also treated as agricultural land cover because of using the grazing lands as farmland in one season of the year. We merged shrimp farms with water bodies as they have similar spectral signatures, and it was difficult to distinguish between them in the images except rivers and creeks as they were distinguishable. We deemed that there is hardly any possibilities of remaining any water bodies in the agricultural field in the satellite images of January which falls within the peak dry season of the study region. We considered the year 1990 as a benchmark time for land use and land cover analysis because it marked a significant period in the post-liberation industrial growth of Bangladesh. This time frame allowed us to capture the changes in land use and land cover over a substantial period, which is crucial for understanding long-term trends and identifying the impacts of industrialization specially evaluating the expansion of shrimp farming.

We collected a total of 150 GPS points (GPSPs) for validating the land-use and land-cover (LULC) classifications of each region. 80% of the GPSPs were used for training the classification and the remaining 20% of the GPSPs for validation purposes. The error matrix was created by assessing user accuracy, producer accuracy, and total accuracy in order to evaluate the accuracy of picture categorization. The accuracy was then assessed by calculating Cohen's Kappa coefficient using Eq. (3).

(3) $$k=\frac{N\sum_{i=1}^{r}X_{ii}-\sum_{i=1}^{r}X_{i+}X_{+i}}{N^{2}-\sum_{i=1}^{r}X_{i+}X_{+i}}$$

Where, N is the number of observations, ‘r’ the number of rows in the error matrix, ‘Xii’ the number of observations in row ‘i’ and column $X_{+i}$ and $X_{i+}$ the marginal totals for row i and column i.

### 2.4 Data analysis

A Probit model was used for computing the maximum likelihood of independent variables affecting livelihood in two moderate and high salinity zones. The dependent variable was binary i.e. coded as 0 and 1 (where 1 for change, true or yes, and 0 for no change, false and no etc.) while independent variables were binary or ordinal.

(4) p=Pr(Y=0)=C+(1C)F(xβ)

In Eq. (4), a vector of parameter estimations is β; F stands for the cumulative distribution function (the normal, logistic, or extreme value); x represents a set of explanatory variables; p represents the likelihood that a response will be given; and C represents the natural (threshold) response rate.

A GPS device was used to obtain the coordinates of ground-truthing points of the study sites. Image processing and mapping were done using ENVI v5.3 and ArcGIS v10.9. For analyzing the land use transition and livelihood change pattern, we used “networkD3” and “ggplot2” packages in R statistical software.

## 3 Results

### 3.1 Changes in land use and land cover from 1990 to 2021

**Fig. 2:**
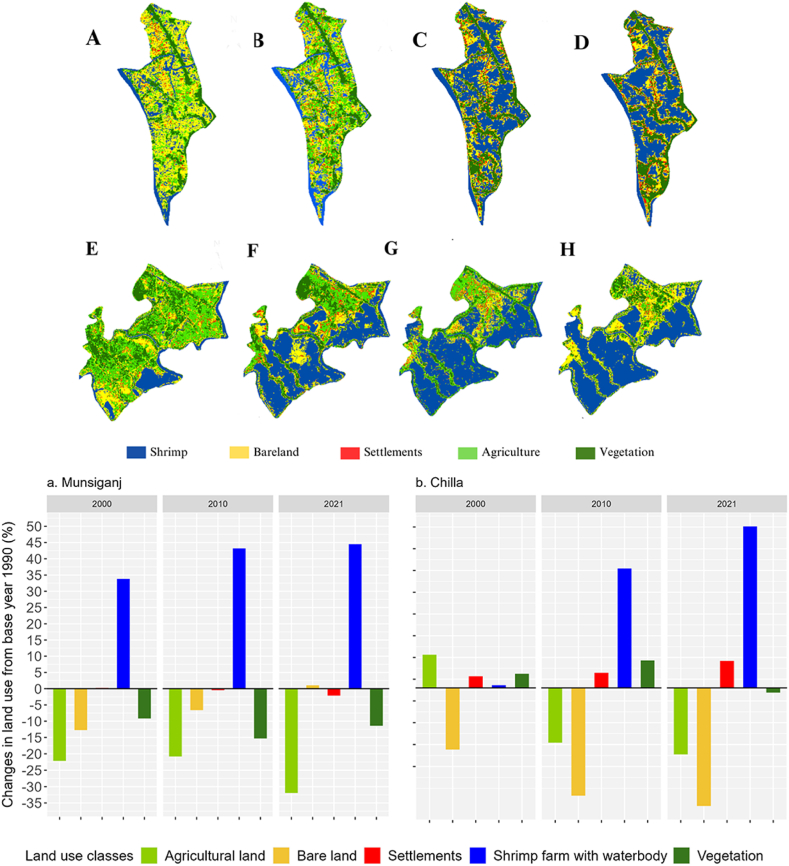
Map showing land use changes in Chila union (MSZ), Satkhira district, for the years 1990, 2000, 2010, and 2021 (as A, B, C, and D, respectively), and in Munshiganj union (HSZ), Bagerhat district, for the same four periods, namely 1990, 2000, 2010, and 2021 (as E, F, G, and H, respectively). Additionally, bar chart showing the different types of land use changes from the base year of 1990.

**Fig. 3:**
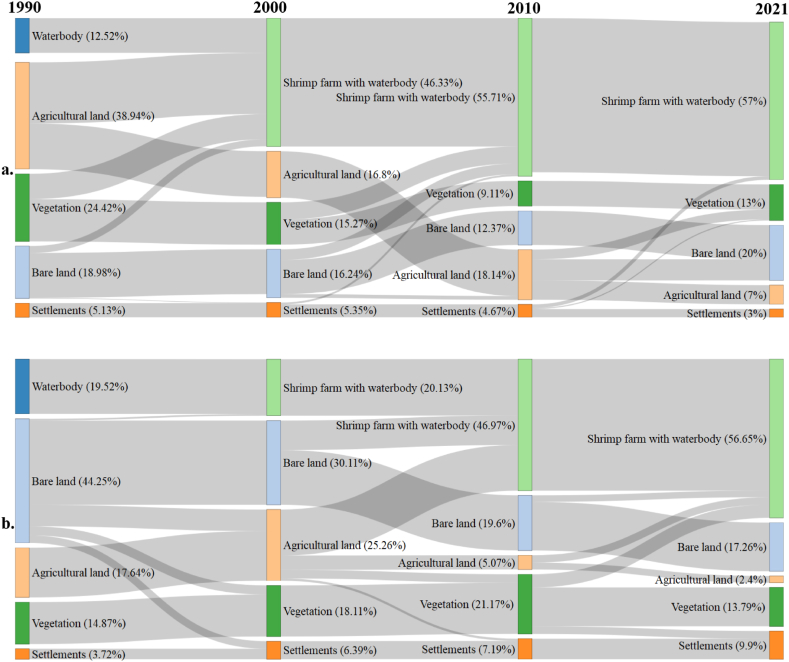
LULC classes change transitions for 1990–2021 of Munsiganj (a) and Chila (b) of Bagerhat and Satkhira districts respectively.

A dramatic alteration of land covers was detected from the analysis of satellite images between 1990 and 2021 that detected five different types of land covers namely shrimp ponds, bare lands, settlements, agricultural lands and vegetation covers (Fig. 2). In 1990, agriculture farming covered 17.64% and 38.94% of areas respectively in MSZ and HSZ while water body was limited to 19.52% in MSZ (Fig. 2 A-D, Fig. 3b) and 12.52% in HSZ (Fig. 2 *E*-H, Fig. 3a). However, the scenarios changed afterwards, the shrimp farming area with water body increased to 37.13% in MSZ of Chila (Figs. 2b) and 44.48% in HSZ of Munshiganj (Fig. 2a) by 2021 compared to the base year 1990. At the same time, agriculture practices, once the most dominant land use in the coastal belts of Bangladesh, had continued to decrease and currently, only a negligible portion is left for agriculture in both HSZ (7%) and MSZ (2.4%) (Fig. 2). The crop cultivation was mostly replaced by shrimp farming in both high and moderate saline-affected areas (Fig. 2, Fig. 3). In 2021, on average, the rise of shrimp farming lands was 41%, settlements were 7% while the fall of agricultural land, vegetation and bare lands were 24%, 13% and 25% respectively compared to the reference year 1990 (Fig. 2a and b). Vegetation cover was severely affected in HSZ where it reduced to almost half from 1990 to 2021, however, in the same period there were no remarkable changes of vegetation cover in MSZ (Fig. 3b). As vegetation cover changes mostly included homesteads and excluded agricultural land in the analysis, their drastic reduction in HSZ indicated the possibility of severe soil salinity that dramatically altered the vegetation covers. The indicated reasoning was also supported by the respondents in the FGDs. Besides, the auspicious consequence was the reduction of bare land, particularly in MSZ (44%) which was converted to shrimp ponds that otherwise be left as unused even though it drastically affects the natural fishing sources as revealed in the FGDs. With respect to the settlements, a contrasting scenario was found in HSZ where it reduced slightly while in MSZ it increased to almost three folds.

The analyses showed that the HSZ had more cropland compared to MSZ (Fig. 3) which is contrasting with the usual assumption. During the field surveys, the respondents informed that shrimp farming industries were developed in HSZ but land conversion for shrimp was more rapid in MSZ (Fig. 3b) than in HSZ (Fig. 3a). Additionally, the shrimp ponds in HSZ were well demarcated with the wide bank, where winter vegetables were grown well. Moreover, high-yielding variety (HYV) of rice tolerant to salinity has got popularization in HSZ compared to MSZ. For instance, respondents added that “BRRI-23, BRRI-28 BRRI-53 and BINA-8 were the HYV rice grew well in Munsigonj (HSZ) than in Chila (MSZ). This suggested the promotion of high-yielding salinity tolerant crops offered a good opportunity to cope with the increasing salinity rather than converting cropland to capital-intensive shrimp farming where many of the marginal farmers were severely affected in terms of livelihoods.

### 3.2 Livelihood changes in the coastal zone of Bangladesh

**Fig. 4:**
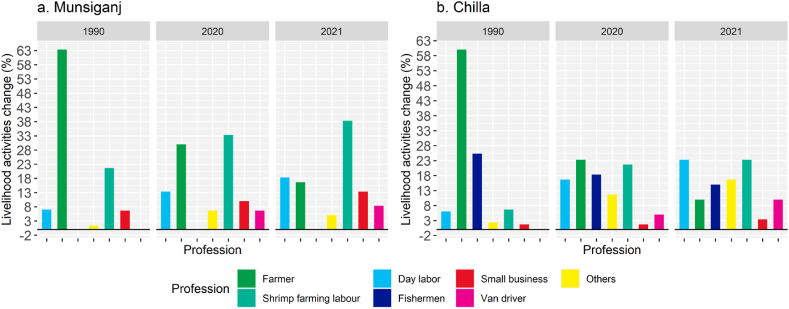
Changes in livelihood patterns in the two study areas in Khulna division from 1990 to 2021 a) Munsiganj, and b) Chilla.

The livelihood patterns were found to be shifted from agriculture to shrimp farming and other labor-oriented jobs over the period of 1990–2021 in the study areas (Fig. 4). The changes in the high salinity zone (Munsiganj) were different from moderate salinity zone (Chilla). Laboure selling in the shrimp farming in HSZ of Munsigonj became almost double (38%) in 2021 compared to 22% in 1990 (Fig. 4a), while it was extended to more than threefold (from 7% to 23%) in the MSZ of Chila between the same periods of time (Fig. 4b). Besides, the occupational structure in the study area as depicted by the upswing of some floating jobs and the decrease of agricultural farmers (−80%) and fishermen (−40%) in HSZ, the two most predominant economic propellers of the pre-salinization period, while the number of farmers reduced by 83% in the same period of time. In addition, not a single person was found to be involved in the traditional fishing profession (except shrimp) in HSZ while in MSZ, and the number fell by 40%.

The local community perceived a number of natural and anthropogenic factors that had a strong link with the livelihood changes. A strong significant linkage was found between livelihood changes with salinity followed by flood and cyclone respectively (Table 3). Salinity was identified by the respondents as the main driving force for inducing such changes in both HSZ and MSZ (Appendix A). Besides the salinity, the respondents opined that cyclone, flood and tidal inundation created water logging conditions and intensifying soil salinity, which creates unfavorable conditions for agricultural crop production and encourages shrimp production, thereby modulating the livelihoods (Appendix A). They also observed that shrimp farming started before 1990s was restricted only to low-lying lands unsuitable for agricultural crops. Ensnaring to the profit, agricultural land was started to convert into shrimp farming, polluting the neighboring land with huge salinization. This turned the land unyielding for crops and it gradually became fallow bare land most of which was successively occupied for shrimp farming by the affluent persons. Family size was identified as the fourth influential factor modulating local livelihood. Increasing the number of family members put more burden on sustenance, consequently played a role in changing the livelihoods. Conversely, a negative relation was found between livelihood changes and education status of the respondents (Table 3), more education in the family means increase of resilience and adaptation capacity.

**Table 3:** Factors driving the livelihood changes in the study areas over the periods from 1990 to 2021.

Key factors affecting livelihoods	Livelihood change (change = 1, no change = 0) of MSZ		Livelihood change (change = 1, no change = 0) of HSZ		Livelihood change (change = 1, no change = 0) taking total 240 respondents	
	MSZ		HSZ		Average weighted	
	Coef.	P (95% CI)	Coef.	P (95% CI)	Coef.	P (95% CI)
Education^c^	−0.08	0.5 (−0.4 to 0.22)	−0.11	0.3 (−0.35 to 0.13)	−0.13	0.1 (−0.3 to 0.04)
Cyclone^a^	0.76	0.1 (−0.2 to 1.72)	0.38	0.22 (−0.24 to 1.0)	0.68	0.007 (0.18–1.19)
Salinity^a^	1.5	0.008 (0.39–2.6)	1.38	0.002 (0.52–2.2)	1.37	0.000 (0.74–2)
Family size^b^	0.21	0.6 (−0.8 to 1.3)	0.22	0.4 (−0.33 to 0.77)	0.36	0.01 (0.08–0.63)
Flood^a^	1.9	0.005 (0.61–3.3)	0.41	0.2 (−0.25 to 1.0)	0.98	0.000 (0.43–1.53)
Constant	−2.97	0.2 (−8 to 2.1)	−2.58	0.1 (−6.33 to 1.17)	−2.78	0.000 (−4.3 to −1.3)

*a, b, c indicates the level of significance in a descending order, same letters indicate the equal significance. ‘Coef’ is the coefficient.

### 3.3 Land use capacity to provide ecosystem services

**Fig. 5:**
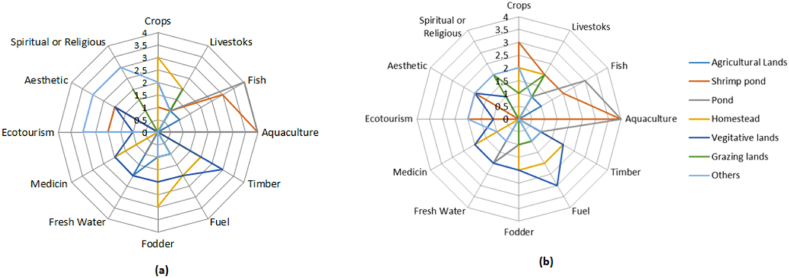
Existing land use and land-cover capacity to provide various ecosystem services in (a) Chila (b) Munsigonj.

A total of 12 indicators of ecosystem services were identified in the existing land use systems in the study area (Fig. 5, Appendix B). It was observed from the FGDs that the respondents were mostly concerned with the provisioning and cultural ES while generally unaware of the regulatory services except for freshwater availability. The respondents of the FGDs opined that both the study regions (both HSZ and MSZ) became suitable for producing shrimp and other sea fishes. Progressive declination of freshwater fish and various vegetable crop productions pushed the local people to rely on the market for those commodities. Among 12 indicators, agricultural land had moderate capacity (2) to provide crops and freshwater regulation, and very low capacity (1) to provide food, fodder, fuel woods, timber, and fish both in MSZ and HSZ (Appendix B). Shrimp farming ponds and other freshwater ponds received a very high score (4) for aquaculture in both MSZ (Fig. 5a) and HSZ (Fig. 5b). Shrimp farming ponds were reported to have a low capacity (1) to produce crops, fodder for livestock and some brackish water fish in MSZ, but in the case of HSZ, it had a moderate capacity (2) to produce crops and fodder (Appendix B and Fig. 5). Homesteads had a high capacity (3) to produce crops both in MSZ and HSZ and a moderate capacity (2) to provide fodder, fuelwood, timber, and medicinal plants (Fig. 5). Grazing lands had a moderate capacity (2) for raring livestock and very low capacity (1) to provide fodder both in MSZ and HSZ. Other fellow lands such as premises of the schools or mosques, roadside etc. Had moderate capacity (2) to provide crops both in MSZ and HSZ and high capacity (3) for recreation and religious aspects in MSZ and a moderate capacity (2) in HSZ (Appendix B).

## 4 Discussion

Land use changes in the coastal regions are very common phenomena owing to increasing river salinity and other natural disasters. The present study investigated the effect of salinity hazards on land cover changes, livelihood, and ecosystem services of the coastal areas in the context of Bangladesh. The study found that salinity intrusion as well as other natural hazards such as floods and cyclones significantly affected the livelihood of coastal adjacent people, and salinity intrusion was identified as the main natural driving force for land use change that greatly affected the ecosystem services. During the flood and tidal inundation, brackish water enters easily into agricultural lands and other areas, creating longtime waterlogging condition as the area is surrounded by a coastal embankment. Thus, most of the area has become unproductive for crop cultivation that resulted in the increase of bare lands. Therefore, a large number of land remarkably shifted to shrimp cultivation, and shrimp farming has increased tremendously in the coastal deltas of Bangladesh over the period of time [5,35]. Besides, longtime water logging also emboldens brackish water shrimp (*bagda*) production [28,36]. Despite the apparent financial benefit of shrimp farming [26], the present study revealed that it is not an effective adaptive strategy or a viable option for the local inhabitants. The higher price of shrimp motivated people to prefer commercial shrimp farming over other crop production; hence the land use patterns were being changed gradually (Fig. 3). The people who do not have enough land and money are reported to be unable to start shrimp farming and their land remains uncultivated due to high salinity. Ultimately, they used to sell or lease their lands to others and consequently they become jobless or shift to other occupations (Fig. 4). In spite of the shrimp sector's contribution to the national economy, a study by Ref. [37] found salinization was strongly associated with shrimp farming as an adaptive strategy that has a negligible impact on reducing poverty among vulnerable and marginalized people living in Bangladesh's coastal areas [38]. The maximum share of the monetary benefits of shrimp farming goes to the outside of the local community [21,39,40]. In fact, local communities are the bearer of the antagonistic social, environmental and economic effects of shrimp farming [7], they only obtain little monetary wages [21,39]. Our findings revealed that the local communities, mostly the agricultural farmers, looked for alternative jobs or became jobless after losing their croplands (Fig. 4). In contrast to the enormous profit of shrimp farming [26], adverse impacts on the traditional coastal livelihoods, ecosystems along with long-term societal and economic prospects were also revealed in our study that support the other studies [3,7,41]. The magnitude of the effect depends on the household characteristics, education, wealth and land ownership [17]. For instance, a household with more capital assets can use the advantages of high salinity by adopting saline aquaculture [42] while the marginal poor farmers are less likely exploit the opportunity [43]. This widens the socio-economic inequalities in the society where marginal farmers are forced to sell their labor leaving the original professions of crop farming or fishing, the irony is that they are employed as a labor force on their own land which is sold or leased to the rich people who converted them to shrimp ponds. The consequence is the increasing vulnerability of the communities to sustain their livelihoods.

In most studies, the effect of the land transformation towards shrimp farming in the context of livelihoods and ecosystem services was missing that has been addressed in present study in a comprehensive way. Agricultural sectors manifestly bear the burden of salinity effect in the study area, it is the key challenging aspect that restricts the production of agricultural crops as also found in other study [44]. The study performed by Ref. [45] manifested the concerns of the poor villager's livelihood affected by environmental changes associated with saltwater intrusion inducing changes in the cultivation system of rice along with other crops to shrimp farming. It is found [46] that a long-time shrimp farming in the same field degrade the soil and make it worst for agricultural farming, thus changing livelihoods. High salinity makes the land unsuitable for agricultural crops and encourages shrimp farming which is capital intensive [47]. Besides salinity, various calamities such as tidal surges, cyclones, freshwater shortages, and floods are also the key driving forces in changing livelihoods (Table 3). Frequent cyclones, high tide, floods, sea-level rise, and poor management of coastal embankments are the regular phenomena that create waterlogging conditions with salinity intrusion in agricultural lands (Appendix A). For instance, two massive cyclones Aila and Sidr made the livelihood most vulnerable in coastal regions of Bangladesh by enormous losses in economical, infrastructural, and agricultural sectors [48]. According to Ref. [49], there is a concern that the increased frequency of tropical cyclones will lower income and living standards in tropical coastal communities.

The driving forces that change the livelihoods of the local communities most likely linked with the ecosystem assets providing various crucial services (Appendix B). In our study, some basic household provisions like fuelwood, food, fodder, and fish were found to be reduced even though shrimp supply increased but it is beyond their means to buy (Fig. 5). The effect on fish and rice production severely affected the sustenance of the communities as they used to rely on indigenous fishes collected from the natural sources [6] and grown rice in the cropland for consumption which later they needed to purchase from the market. Decreasing grazing land and fodder adversely affected the livestock rearing that ultimately affected their dietary nutrition supply and income. The results are in line with a study by Ref. [50], that the salinity of coastal Bangladesh's grazing terrain significantly affects livestock rearing. Earlier study [7] found the local population in Bangladesh's coastal regions is becoming more vulnerable as a result of a decline in ecosystem services. The reduction of ecosystem services caused by salinity in the coastal districts of Bangladesh, particularly the availability and quality of water and land stability found to deteriorate in a study reported by Ref. [9] that corroborated our present study in a sense that overall service potential of the ecosystems is at a stake that warrants urgent attention to ameliorate. Most of the previous studies focuses on the causative link or indicators of changes ES, particularly the food and drinking water, few studies attempted to link the change of ES with the livelihood, LULC change or finding out the driving force of changing the ES. The present findings have added significant evidence that changes of coastal ES services are associated with the LULC that was driven predominantly by salinity intrusion. The decrease of ES services is not uniform in our two study regions. For instance, MSZ was found in a better position in serving religious, aesthetic and ecotourism and fish (not shrimp) production services in compared to HSZ while crops, timber, fuel are comparatively higher in HSZ (Appendix B). In HSZ, many timber species adopted to high salinity have been observed to grow as well as salinity resistant agricultural crops are favored by the farmers. Therefore, in the policy making process, it should be taken into consideration which services are adversely affected or which one is promoted in the changing scenario of ecosystem. We employed participatory assessment methods to evaluate the services that are directly provided to the community by the ES. Thus, the findings of the ES services represent the perceptions of the local community, has potential implications in policy making processes.

Salinity pollution cannot be fully eradicated from the study areas (47,201 km^2^ coastal areas) as the country is located at very low altitudes on the shoreline of the Bay of Bengal having a coastal area of which is about 1.5 m above the mean sea level [[51], [52], [53]]. Nevertheless, management aspects should get the priority to minimize the impact and to decrease the anthropogenic effects of salinity pollution. The vulnerability of the coastal zone has increased due to anthropogenic disturbances and climate change [28,[54], [55], [56], [57]]. The problem of salinity pollution is not limited to Bangladesh only, it is a major environmental problem affecting many deltaic countries [[58], [59], [60]] adversely affecting 20% of the agricultural land and 50% of the total cropland globally [35]. Productivity of agricultural crops, mainly vegetable crops that show low tolerance to salinity might be affected largely by soil salinity. By 2050, it is predicted that soil salinity will damage nearly 50% of the world's arable land [61]. In this context, the implication of findings is not limited to the local scale, rather it is applicable to other coastal areas of the developing countries.

## 5 Conclusion

The present study manifested the argumentative impact of salinity which has influenced many aspects of people's livelihoods and occupations by altering land uses, and ecosystem services. It is evident from the study that the livelihood of the people became more burdensome with the increasing shocks of salinity. The most conspicuous effect was on the agricultural sectors, the life blood of the rural economy of the country. Our study found the increase of the substantial amount of land for shrimp farming has reduced the agricultural farming professions which is tantamount to the ‘double-edge sword’. The increased labor-oriented job seekers suggesting the downgrading of the poor to poorer. Aside from the marginalizing effect on the poor farmers, the study also found a huge ecological impact in the context of ecosystem services. Despite the widespread use of shrimp farming as an adaptive approach in coastal areas, respondents believed that it was less likely to be successful in eradicating poverty there. The introduction of shrimp farming scarcely delivered prosperity to the rural poor in the study area, as implied by the current trend of shifting occupations to labor-oriented jobs and reducing agricultural land. The salinity of the area, which is considered to be the primary stressor of the farm-based livelihood, was made worse by the conversion of land to shrimp farming. The livelihoods of the rural population have been drastically altered by the shrinking of agricultural area and the bareness of the soil. The majority of the locals blamed manmade factors, such as the conversion of land for shrimp farming, for the salinity's rise.

While the findings provide valuable insights into the drastic transformation in land use systems from 1990 to 2021 and how these changes have exacerbated the occupations and livelihoods of the community, it does not address other factors that may also impact these systems, such as climate change or economic development policies. The strength of the study is evaluating LULC changes and perceptions of the local community in the field survey that reflects the real scenario of the current issues. The insights of the study can inform policy decisions aimed at increasing community resilience. Additionally, this study contributes to our understanding of how salinity affects rural livelihoods and ecosystem services in coastal areas, which is an important area of research given the increasing threat of climate change on these systems. However, the study did not explore potential solutions or interventions to mitigate the negative impacts of salinity on rural livelihoods and ecosystem services. Future research could investigate effective strategies for addressing the challenges to mitigate the salinity hazard in the coastal areas to uplift the rural livelihoods. In the present context, it is urgently needed to formulate and implement the appropriate strategic plans to slacken the negative impact on ecosystems and enhance human well-being. A careful and judicious thought should be given before converting the traditional agricultural land to shrimp aquaculture to minimize the salinization and to ensure the ecosystem services for the subsistence of the local community.

## Funding

The work was funded by 10.13039/501100007944Shahjalal University of Science and Technology Research Center (Grant number: MS04/2017–18).

## Author contribution statement

Rofiqul Islam, Romel Ahmed: Conceived and designed the experiments, wrote the paper, performed the experiments.

Biplob Dey: Analyzed and interpreted the data; contributed reagents, materials, analysis tools or data, wrote the paper.

Saiful Haque: Performed the experiments, wrote the paper. Sokina Aktar, Md. Saifuzzaman Bhuiyan, Mohammad.

Saidul Arif: Analyzed and interpreted the data; performed the experiments. Md. Ahosan Habib Ador, Mohammed.

Masum Ul Haque, Narayan Saha: Contributed reagents, materials, analysis tools or data.

### Data availability statement

Data will be made available on request.

## Additional information

Supplementary content related to this article has been published online at [URL].

## Declaration of competing interest

The authors have no significant competing financial, professional or personal interests that might have influenced the performance or presentation of work described in this manuscript.

## Figures and Tables

**Figure:**
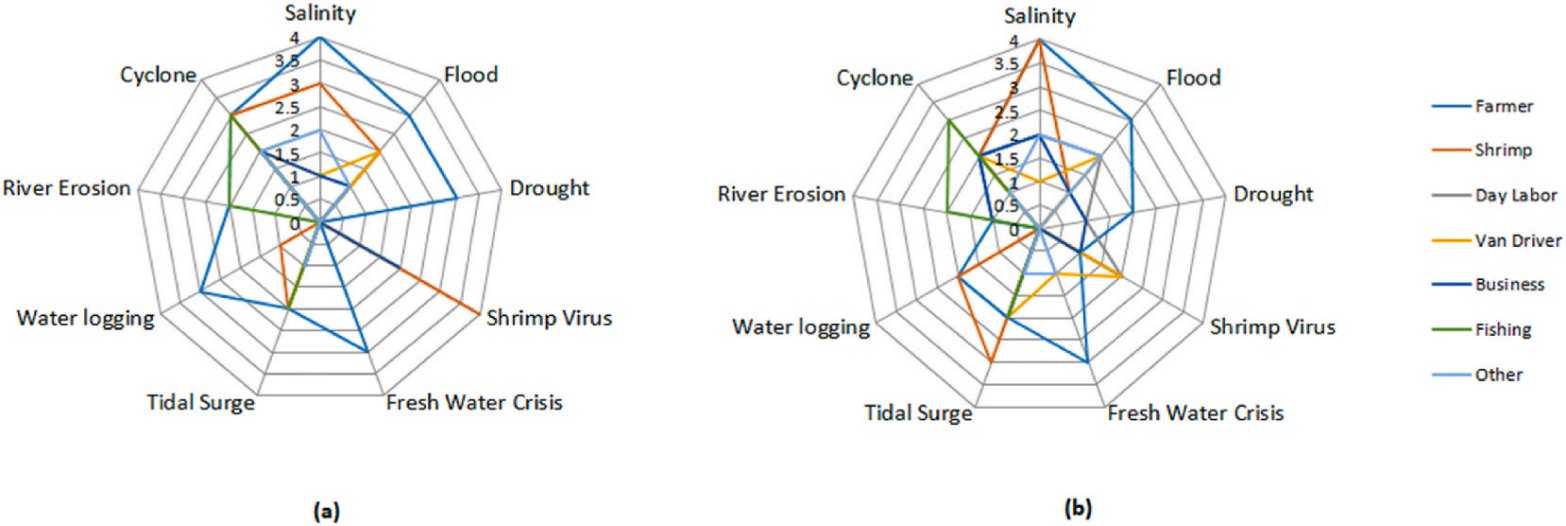


**Table 01:**
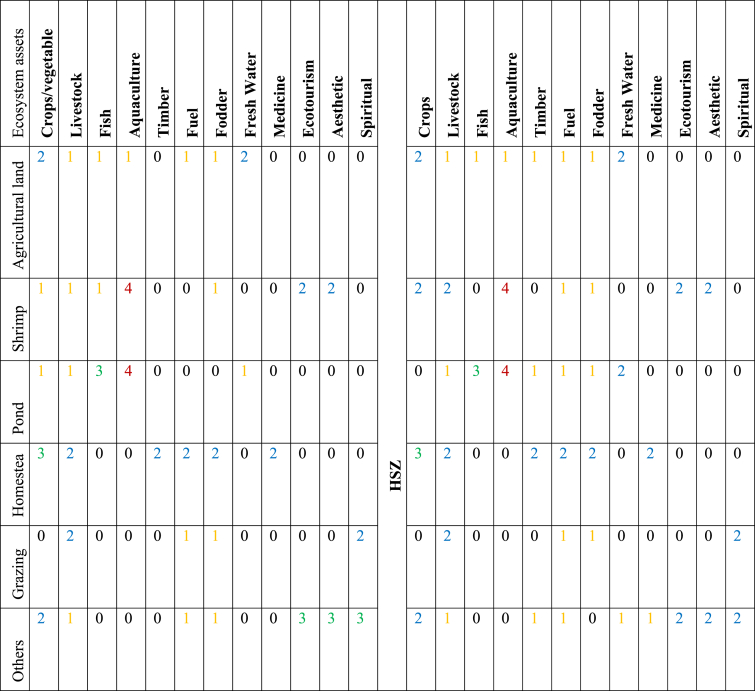
Relative capacity of present land use to provide Ecosystem Services in MSZ (Chila) and HSZ (Munsigonj). Values indicate the relative capacity; 4 = Very high capacity; 3 = High capacity; 2 = Moderate capacity; 1 = Low capacity and 0 = no capacity.

**Table 02:** Accuracy matrix of land use and land cover changes that were evaluated for this study.

	Munshigonj		Chila	
	User accuracy	Producer accuracy	User accuracy	Producer accuracy
Shrimp farm	1.00	1.00	0.83	1.00
Bare land	0.67	0.80	0.83	0.83
Settlements	1.00	0.86	1.00	0.88
Agricultural land	0.67	0.67	0.67	0.80
Vegetation	0.83	0.83	0.83	0.71
Overall accuracy	83.33%		86.67%	
Kappa	79.17%		83.00%	

## Appendix A

Various natural and anthropogenic disasters impacting the livelihoods of the respondents in HSZ of Munshigonj (a) and MSZ of Chilla (b) identified in Focus group Discussions (FGDs).
