# Supplementary material for: Salinity hazard drives the alteration of occupation, land use and ecosystem service in the coastal areas: Evidence from the south-western coastal region of Bangladesh
Source: Heliyon. 2023 Jul 21;9(8):e18512. doi: 10.1016/j.heliyon.2023.e18512 (PMC10413002; doi:10.1016/j.heliyon.2023.e18512)
Supplement: Multimedia component 1 [file mmc1.docx]

**Questionnaire on**

**Salinity effect on occupation, land use and ecosystem service in the coastal area**

Interview of Respondent

General Information:

Name:

Age: Sex:

Union: Village:

Education Level:

Livelihood:

Q: What is / are main source of income?

| Present | | 10years back(2005) | | 20years(1995) | 1990 | |
| --- | --- | --- | --- | --- | --- | --- |
| Crops production |  |  |  |  |  |  |
| Shrimp |  |  |  |  |  |  |
| Labor or share crops |  |  |  |  |  |  |
| Other(Name) | |  | |  |  | |

Q: No of family member and earning person.

Ans:

Q: Average family income………..

Q: For how long such type of change (livelihood) (if any) occurs?

Ans:

Q: What is / are the driven factors for such change?

| Types | Yes(1) or No(0) | Types | Yes(1) or No(0) |
| --- | --- | --- | --- |
| Cyclone |  | Financial cause |  |
| Salinity |  | Social |  |
| Flood |  | Political |  |
| Tidal Surge |  | Other Pressure |  |

Q: How does the changes affect the income and others?

Ans:

Q: What types of adaptive mechanism they applied?

Ans:

Q: How long the shrimp industries developed here?

Ans:

Q: Whether they forced to become a labor?

Ans:

Q: Are they have own any shrimp pond?

Ans:

Q: How much the get for working shrimp pond?

Ans:

Land Use Change

Land use:

1. Present land holdings ………………..
2. Your fathers or grandfathers land holdings…………………………
3. Why did you sell lands? (If Present land is fewer)……………………………………..
4. Amount of land

| Types(2016) | Ha or % | Types (2000) | Ha or % | Types(1990) | Ha or % |
| --- | --- | --- | --- | --- | --- |
| Agriculture |  | Agriculture |  | Agriculture |  |
| Homestead |  | Homestead |  | Homestead |  |
| Pond |  | Pond |  | Pond |  |
| Others |  | Others |  | Others |  |

1. No. of your children ……………………………………..

Q: What types of land use change recently?

Q: Is the change is very recent or for how long?

Ans:

Q: What types of conversion is common?

Ans:

Q: What types of crops (both vegetable and woody plant) are gown in homestead or surrounding at present?

Ans:

Q: Is there any changes then previous?

Ans:

Q: If, then what types?

Ans:

Q: What are the possible driving factors of land use changes?

Ans:

Q: How do they adopt with new types of crops?

Ans:

Q: How did the shrimp culture affects?

Ans:

Q: How many shrimp pond and/ or are there?

Ans:

Q: Did they acquire the land from the local people bye forced or at fair price?

Ans:

Q: Did they encroach to the govt. or forest land?

Ans:

**Ecosystem Service:**

1. Existing ecosystems of the locality

| Types | Amount in ha or % | Types | Amount in ha or % |
| --- | --- | --- | --- |
| Natural vegetation |  | Homestead |  |
| Plantation |  | Agri or crops land |  |
| Pond or other aquatic |  | Common land |  |
| Shrimp pond |  | Grazing land |  |

1. Types of ecosystem services they get

| Provisioning | | | Supporting or regulating | | Cultural | |
| --- | --- | --- | --- | --- | --- | --- |
| Food | Types | Amount | Types | Yes or not | Types |  |
|  | Crops |  | Soil formation |  | Spiritual or religious values |  |
|  | Livestock |  | Local climate regulation |  |  |  |
|  | Fish |  | Erosion control |  | Aesthetic Values |  |
|  | Aquaculture |  | Water regulation |  | Ecotourism |  |
|  | Wild food |  | Supporting from Natural hazards |  |  |  |
| Fiber | Timber |  |  |  |  |  |
|  | Fuel |  |  |  |  |  |
|  | Fodder |  |  |  |  |  |
|  | others |  |  |  |  |  |
| Medicine |  | |  |  |  |  |
| Freshwater |  | |  |  |  |  |

Q: How did they depend on ecosystem?

Ans:

Q: How frequent they collect fodder, fuel, fishes etc.?

Ans:

Q: How they depend on ecosystem? For daily need or for livelihood?

Q: What amount of these (fuel wood, fodder, timber etc.) they collect?

Ans:

Q: is the fuel or fodder available in surrounding or not?

Ans:

Q: How much time you spend to collect fuel or fodder?
Ans:

Q: How many cattle (cows and goats) do you have?

Ans:

Q: (if no cattle or very few then why) is there have available land for grazing or source of fodder?

Ans:

Q: How much time do you spend to collect fodder?

Ans:

Q: Approximately how much more time need in compare to 10 or 20yrs ago?

Ans:

Q: Why does the amount of fodder and/ or grazing land decreased? (Opinion)

Ans:

Q: Do you have available fresh water for drinking?

Ans:

Q: From where do you collect it?

Ans:

Q: (if not from tube-well of his own) How much time do you spend to collect water?

Ans:

Q: How far the source from your house?

Ans:

Q: Approximately how much more time needs to collect water in compare to 10 or 20yrs ago?

Ans:

Q: Why the sources of drinking water are not available now?

Ans:

Q: Did the Govt. or other organization help them for any alternative?

Ans:

Q: What types of factors (natural and anthropogenic) affecting ES………………..

|  |  |  |
| --- | --- | --- |
|  |  |  |
|  |  |  |
|  |  |  |

What are available plant or vegetable cops in this area? What are saline resistant variety (tree, shrubs, crop)? What are flood resistanr variety?

How many live stock do u have, what are they (cow, goat, buffalo), what the eat? From where u get those? Do u buy or collect? Is there any land for grazing? What type of those land? Do they sufficient?

If u don’t have livestock, why not? U donk like or u have no time or no fodder?

Do you have poultry, how many, how do u coolect their food? Do u buy? How much you earn from the poultry, if u eat, how many you ate last year? What is the equivalent price of those of you buy?

Do you have vegetable garden? Where (cropland or home stead? Or roof top, or floating garden? ) how much u consume and how much u sell? If you buy from the market, how much u need to pay? Do u cultivate winter vegetable at your home garden? Is there any special measure to cultivate those? If not cultivating, why (because u r landless, no space, not growing due to salinity? )
